# Nutritional Composition and Digestibility of Corn Ethanol Coproducts in Swine Feed: A Systematic Literature Review

**DOI:** 10.3390/ani16040560

**Published:** 2026-02-11

**Authors:** Vivian Luana Rothmund, Charles Kiefer, Ana Paula Silva Ton, Maicon Sbardella, Vinicius Ricardo Cambito de Paula, Luana Cardoso Cirino, Igor Campos Lima, André Luiz Barros Leda, Manuela Maria de Moura, Nataly Stanghilin Pissinatti, Jessica Ferreira da Silva, Leonardo Willian de Freitas, Anderson Corassa

**Affiliations:** 1 Agrarian and Environmental Sciences Institute, Federal University of Mato Grosso, Sinop 78.550-000, MT, Brazil; ana.ton@ufmt.br (A.P.S.T.); maicon.sbardella@ufmt.br (M.S.); vinicius.paula2@ufmt.br (V.R.C.d.P.); luanacardosocirino@gmail.com (L.C.C.); jonessouza671@gmail.com (I.C.L.); andreluiz.biso@gmail.com (A.L.B.L.); manuelamariamoura20@gmail.com (M.M.d.M.); natalypissinatti@gmail.com (N.S.P.); jessica.silva10@sou.ufmt.br (J.F.d.S.); lwillianf86@gmail.com (L.W.d.F.); 2 Veterinary and Animal Science Department, Federal University of Mato Grosso do Sul, Campo Grande 79.070-900, MS, Brazil; charles.kiefer@ufms.br

**Keywords:** alternative ingredients, database, digestibility, energy, pig nutrition

## Abstract

Corn ethanol coproducts contain high concentrations of protein, lipids, and fiber; however, their nutritional composition and digestibility vary significantly across manufacturing processes. This study aimed to survey and synthesize literature data on corn ethanol coproducts and their effects on pig digestibility. A systematic search was conducted between August 2023 and October 2025 across databases, including Periódicos Capes, ScienceDirect, Web of Science, Scielo, and Google Scholar. The search targeted publications from 2010–2005 using keywords related to DDGS, pigs, energy, and amino acids. Among the 177 articles meeting the inclusion criteria, corn DDGS was the most common coproduct (359 observations), followed by wheat and sorghum. The results indicated that wheat DDGS had the highest protein and phosphorus contents, whereas corn DDGS presented higher crude fiber and NDF levels. Furthermore, corn DDGS provided the highest digestible and metabolizable energy values, with amino acid digestibility coefficients generally surpassing those of other sources. This updated database serves as a vital tool for improving the predictability of these ingredients in swine nutrition.

## Simple Summary

The objective of this study was to conduct a literature review on the composition of ethanol coproducts and their effects on pig digestibility. The raw materials most frequently used for dried distillers’ grains with solubles (DDGS) production were corn (*n* = 359), wheat (*n* = 32), and sorghum (*n* = 15), whereas only corn was used for the production of high protein distillers’ grains (HPDDGs) (*n* = 31) and high protein distillers’ grains with solubles (HPDDGS) (*n* = 14). The composition of the corn ethanol coproducts varied over the analyzed years. The data provide a broad analysis of the variability in the composition and energy values of the coproducts, as well as their digestibility. The collected data reinforce the importance of considering the high variability of corn ethanol coproducts in pig diet formulation.

## 1. Introduction

Concern about the consequences of the greenhouse effect and environmental pollution has driven the expansion of biofuel production. These products represent a viable alternative for reducing the reliance on nonrenewable energy sources. Among biofuels, ethanol has gained prominence in Brazil and worldwide, with sugarcane and corn as the main feedstocks.

The United States is the world’s largest producer and exporter of ethanol, accounting for approximately 52% of global production. In 2024, the country exported approximately 58.2 million liters of ethanol, with Canada, the United Kingdom, and the European Union as its main importers. Brazil ranks as the second-largest ethanol producer globally, contributing approximately 29% of total world production in 2024, followed by India, the European Union, and China [1].

During the 2024/25 harvest season, Brazil produced approximately 37.2 billion liters of ethanol. Approximately 7.8 billion liters (32.4% of total production) of this total originated from plants that use corn as the primary feedstock [2].

In this context, the corn ethanol industry has shown significant growth in Brazil and globally, generating large quantities of coproducts. In 2025, Brazil produced approximately 2.72 million tonnes of ethanol coproducts, with an estimated 2.9 million tonnes projected for 2026 [3]. These coproducts are rich in nutrients and have been widely used in livestock feeding. For swine, dried distillers’ grains with solubles (DDGS) can be included across all production stages, provided that appropriate dietary inclusion limits are met [4]. In 2024, approximately 22% of the distillers’ grains produced in the United States were utilized in swine diets [5].

Among the coproducts of corn ethanol, dried distillers’ grains with solubles (DDGS) have high concentrations of protein, lipids, and fiber, reaching levels up to three times those of corn. However, its nutritional composition and digestibility may vary significantly and are influenced by factors such as the manufacturing process and the raw materials used by the ethanol industry [6,7,8,9,10,11].

To ensure the efficient and safe use of corn ethanol coproducts in animal nutrition, understanding their compositional variability is essential. These variations directly affect diet formulations and require rigorous controls and reliable databases to allow comparisons between different batches and coproduct sources.

The systematic analysis of publications allows organizations to organize data in a critical way, integrating the evidence published in a given area and leading to results with less bias and higher quality, which contributes significantly to the advancement of research. In this context, the construction of an updated database containing the composition of corn ethanol coproducts has emerged as a valuable tool for researchers and nutritionists. This resource can aid in the comparison of different sources of coproducts, in decision-making, and in the formulation of more accurate diets, in addition to making the effects of the use of these ingredients more predictable. The availability of a database can thus make the process of evaluating and choosing ingredients more dynamic and assertive.

Although they provide essential information on feed ingredients, databases commonly used in swine diet formulations, such as the NRC [12] and the Rostagno tables [13], present limitations when applied to corn ethanol coproducts. The NRC [12] is predominantly based on data generated in the United States, reflecting specific feedstock characteristics and industrial processing conditions. In contrast, the Rostagno tables [13], while representative of the Brazilian context, are still derived from a limited number of studies involving these coproducts.

In this context, the development of a database through a systematic literature review covering the 2010–2025 period allows the integration of both national and international evidence, the capture of nutritional variability among these coproducts, and the provision of more comprehensive and up-to-date information. This approach has substantial practical relevance for the swine nutrition industry in Brazil and worldwide.

Therefore, the objective of this study was to survey and synthesize data from the literature on corn ethanol coproducts and their effects on nutrient digestibility in pigs. Specifically, this study aimed to evaluate variations in the nutritional composition of dried distillers’ grains with solubles (DDGS) derived from different feedstocks (corn, wheat, and sorghum), compare differences in digestible and metabolizable energy values between high-protein coproducts, and examine the relationships between metabolizable energy and the main nutritional components of corn DDGS.

## 2. Materials and Methods

### 2.1. Systematic Review

The searches were conducted from 16 August to 28 September 2023, and were updated from 23 September to 9 October 2025, on the platforms Periódicos Capes (https://www.periodicos.capes.gov.br/, accessed on 23 September to 9 October 2025), PubMed (https://pubmed.ncbi.nlm.nih.gov/, accessed on 23 September to 9 October 2025), ScienceDirect (https://www.sciencedirect.com/, accessed on 23 September to 9 October 2025), Web of Science (https://clarivate.com/products/scientific-and-academic-research/research-discovery-and-workflow-solutions/webofscience-platform/, accessed on 23 September to 9 October 2025), Scielo (https://www.scielo.br/, accessed on 23 September to 9 October 2025), and Google Scholar (https://scholar.google.com/?hl=pt-BR, accessed on 23 September to 9 October 2025).

A literature search was conducted to identify studies evaluating the nutritional composition and digestibility of corn ethanol coproducts in pigs. The following keyword combinations were used: “DDGS” AND “pigs” AND “digestibility”, “DDGS” AND “pigs” AND “energy”, and “DDGS” AND “pigs” AND “amino acids”. To identify eligible articles during screening, synonyms were included in the search strategy (DDGS, distillers’ grains, HPDDG, HPDDGS, pig, pigs, swine, digestibility, digestible energy, metabolizable energy, amino acid). The search strategies were adapted to each database via Boolean operators. Specific filters were applied to identify publications dated between 2010 and 2025, and no language restrictions were imposed. The last search was conducted on 9 October 2025.

The initial screening of articles was performed on the basis of titles and abstracts. Studies that did not meet the inclusion criteria were excluded. Theses, dissertations, abstracts, undergraduate final projects, meta-analyses, duplicate articles, studies evaluating species other than pigs, studies lacking nutritional composition and/or digestibility data for the coproducts, and studies involving coproducts derived from processing methods unrelated to ethanol production were not considered eligible. The screening and eligibility assessment were independently conducted by at least two reviewers. Any disagreements regarding study selection were resolved through discussion until consensus was reached.

Data extraction was performed via a standardized Excel spreadsheet. Data were extracted by at least two researchers and subsequently reviewed by an independent researcher. Owing to the descriptive nature of this review, all studies containing data on nutritional composition and/or digestibility were included, even when one or more specific parameters were not reported.

### 2.2. Inclusion Criteria

The inclusion criteria were (1) the use of pigs, (2) the use of dry distillers’ grains with solubles (DDGS), high protein distillers’ dried grains (HPDDGs), high protein distillers’ dried grains with solubles (HPDDGS), (3) the composition and/or digestibility of coproducts, (4) scientific articles, and (5) articles published between 2010 and 2025.

### 2.3. Inclusion of Data

**Figure 1:**
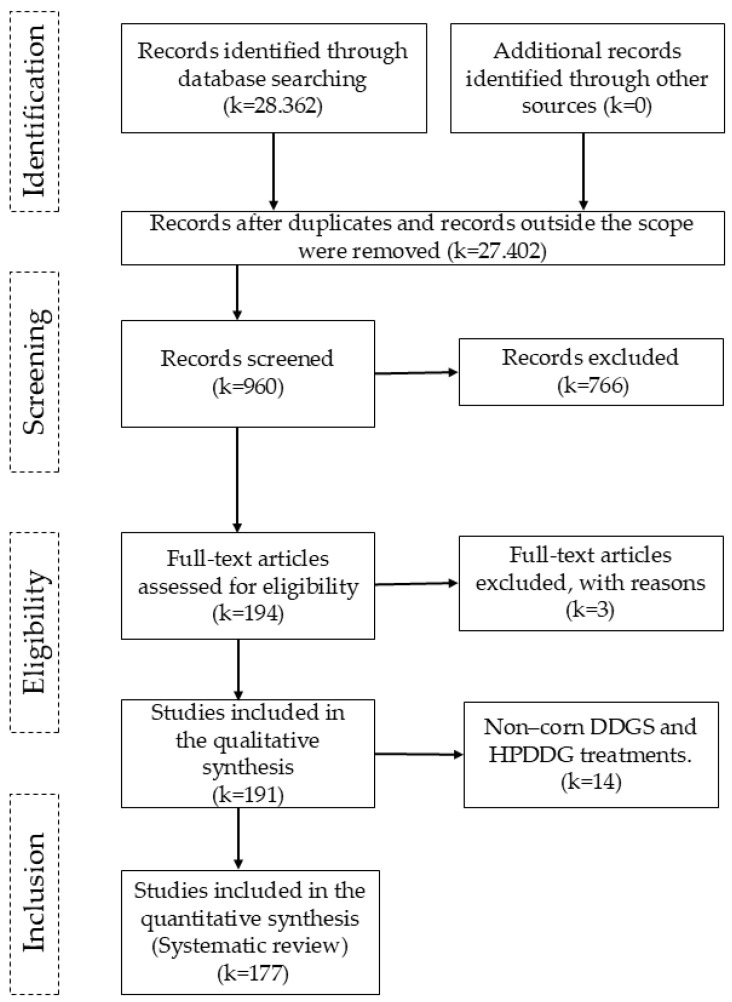
PRISMA flow diagram describing the study selection process. *k* represents the number of articles in each step.

Initially, 28,362 articles were identified through searches in electronic databases. After removing duplicates and articles that did not meet any inclusion criteria, 960 records were screened. During screening, 766 records were excluded on the basis of titles and abstracts; articles with other animals, meta-analyses, theses, or dissertations were excluded, leaving 194 complete articles for eligibility evaluation. Three articles were excluded because they contained coproducts with high moisture content (>30%). A total of 191 articles were considered eligible for inclusion in the database, as they met the preestablished criteria. Finally, 14 articles were excluded because they contained data from other grains or other coprod-ucts that were not high protein distiller’s grains (HPDDGs) or DDGSs, leaving 177 articles for in-clusion in this systematic review [6,7,10,11,14,15,16,17,18,19,20,21,22,23,24,25,26,27,28,29,30,31,32,33,34,35,36,37,38,39,40,41,42,43,44,45,46,47,48,49,50,51,52,53,54,55,56,57,58,59,60,61,62,63,64,65,66,67,68,69,70,71,72,73,74,75,76,77,78,79,80,81,82,83,84,85,86,87,88,89,90,91,92,93,94,95,96,97,98,99,100,101,102,103,104,105,106,107,108,109,110,111,112,113,114,115,116,117,118,119,120,121,122,123,124,125,126,127,128,129,130,131,132,133,134,135,136,137,138,139,140,141,142,143,144,145,146,147,148,149,150,151,152,153,154,155,156,157,158,159,160,161,162,163,164,165,166,167,168,169,170,171,172,173,174,175,176,177,178,179,180,181,182,183,184,185,186] (Figure 1).

The selected articles were identified by author, year, title, DOI, and search platform. The following information was collected from the articles: coproduct, DDGS raw material cereal, initial weight of the animals, DDGS level, bromatological composition data, amino acid, mineral, digestibility values of the coproducts, and method of determination of digestibility.

### 2.4. Statistical Analysis

The variables were analyzed via descriptive statistics with the Statistical Analysis System in Demand for Academics software (Release 3.1.0, SAS Institute Inc., Cary, NC, USA). Parameters such as the mean, coefficient of variation, standard deviation, and maximum and minimum values were calculated for each variable. These parameters were selected to facilitate comparisons with published scientific studies and established feed composition databases. A meta-analysis was not performed due to the high heterogeneity among studies with respect to coproduct types, processing methods, and reported variables.

The analysis was stratified by the coproduct type class and grain source used, allowing us to evaluate the nutritional characteristics and digestibility, as well as the amino acids and minerals in each coproduct. No analyses were performed on the basis of production processes or geographic regions. The composition data, digestible contents, and digestibility coefficients were expressed as percentages (%), whereas the energy values were standardized to kcal/kg. The values indicated by “–” denote incomplete proximate composition data in the tabulated articles. All the data were converted to a dry matter basis.

### 2.5. Risk of Bias Assessment

No assessment of publication bias was conducted, and no formal risk-of-bias evaluation was performed for the included studies. This decision was made owing to the descriptive nature of the review and the substantial diversity observed in experimental designs, methodologies, and the manner in which results were reported across studies. The data were analyzed via descriptive statistics, including means, minimum and maximum values, standard deviations, and coefficients of variation. This limitation was considered when the findings were interpreted. This systematic review was not registered in a protocol database.

## 3. Results

### 3.1. Nutritional Value of Corn Ethanol Coproducts

The raw materials most frequently used to produce DDGS were corn (*n* = 359), wheat (*n* = 32), and sorghum (*n* = 15), whereas only corn was used for the production of HPDDG (*n* = 31) and HPDDGS (*n* = 14), and *n* represents the number of observations extracted from the literature.

The main variables analyzed were chemical composition, energy values, and amino acid profiles, as well as digestibility coefficients. However, not all studies reported these variables comprehensively. Therefore, the number of observations varied according to the variable analyzed. Although some variables had a low number of observations, they were still included in the results to characterize the bromatological composition of these ingredients and to highlight the availability of information in the literature.

As expected, the coproduct chemical compositions were highly varied (Table 1 and Table 2). The mean gross energy (GE) of corn was 4947 kcal/kg, with a minimum of 3295 kcal/kg and a maximum of 6261 kcal/kg, ±448 kcal/kg. The mean DDGS of sorghum was 4812 kcal/kg ± 364 kcal/kg, and that of wheat was 4953 kcal/kg ± 234 kcal/kg, with less variability than the DDGS of corn. In terms of the CP content, the DDGS of wheat was the highest, at 36.28%, and ranged from 26.94 to 46.3%, in contrast to those of corn (30.56%) and sorghum (31.49%) (Table 1).

The crude fiber (CF) content was greater in corn DDGS, with an average value of 9.95%, whereas the average values for sorghum and wheat were 8.75% and 7.79%, respectively. The NDF content also varied, with averages of 35.51% for corn, 40.84% for sorghum, and 31.53% for wheat, indicating that corn and sorghum have relatively high fiber content and structure compared with wheat.

The phosphorus (P) content was greater in wheat DDGS, with a mean of 0.82%, whereas the mean phosphorus (P) content was 0.81 and 0.68% in corn and sorghum DDGS, respectively. The calcium (Ca) concentration was greater in sorghum DDGS (0.18%) than in corn (0.10%) and wheat (0.11%).

In terms of amino acid levels, lysine levels were higher in wheat DDGS, with a mean of 0.99 % ± 0.50 %, whereas those in corn and sorghum DDGS were 0.96 and 0.91%, respectively. The other amino acids presented similar patterns, with higher levels in wheat DDGS than in the other sources.

The HPDDGS and HPDDG included only one source of grain, corn. The GE contents of the HPDDG were higher than those of the HPDDGS, with approximately 5446 kcal/kg and 5040 kcal/kg of GE in its composition. In terms of protein content, the HPDDGS had relatively high levels, with an average CP of 42.50% (Table 2).

The levels of NDF and ADF were lower in the HPDDGS (33.19% and 16.29%) than in the HPDDG (39.90% and 20.69%). Both coproducts showed great variation in their composition, demonstrating discrepant minimum and maximum levels.

The P contents of the HPDDG were higher than those of the HPDDGS, with means of 0.52% and 0.49%, respectively. For Ca, the inverse was observed, where the mean of the HPDDGS was 0.09. The levels of amino acids (AAs) in the coproducts were similar, with no major differences in their composition (Table 2).

### 3.2. Digestibility of Corn Ethanol Coproducts in Pig Diets

Digestibility coefficients (DC) and the energy values of the DDGS of different grains over the years analyzed (Table 3). Compared with the other sources, sorghum had the best dry matter digestibility coefficient, with a mean of 69.25%, while the dry matter digestibility coefficients of corn and wheat were 64.79 and 68.20%, respectively. The DCCP was 71.44% for corn on average, 63.43% for sorghum on average, and 67.78% for wheat.

Sorghum had the best digestibility (65.17%) for DCNDF, while that of corn was 55.59% on average. For DCADF, corn was superior, with a mean of 46.60%, compared with sorghum, which had a mean of 13.83%. No data for these variables were reported for wheat.

Compared with the other sources, corn DDGS had higher digestible energy (DE) and metabolizable energy (ME) values, with 3566 kcal/kg (*n*: 86) and 3307 kcal/kg (*n*: 81), respectively. Sorghum had DE and ME values of 3349 kcal/kg and 3156 kcal/kg, respectively. No DE or ME data for wheat were reported. The digestibility of gross energy was similar for corn and sorghum, with values of 67.09 and 66.55%, respectively, whereas wheat had a DCGE value of 75%.

In terms of the coefficients of digestibility of amino acids and digestible amino acids, the coproducts from corn presented, in general, higher values than those from the other sources (Table 4).

Compared with the HPDDG, the HPDDGS had a greater DCGE, with a difference of approximately 5% between the levels. The other variables were not observed in the selected articles (Table 5).

In terms of DE and ME, the HPDDGS presented contents of 3628 and 3545 kcal/kg, respectively, with a low number of observations. In contrast, with a greater number of observations, compared with the HPDDGS, the HPDDG had higher DE and ME values, with values of 3653 and 3375 kcal/kg, respectively.

With respect to the digestibility coefficients of amino acids, HPDDGS generally presented higher values and was more digestible than HPDDG. The same can be observed for the digestible amino acid content, where HPDDGS resulted in a higher digestible amino acid content than HPDDG did, although the levels of amino acids in the composition were similar (Table 6).

**Figure 2:**
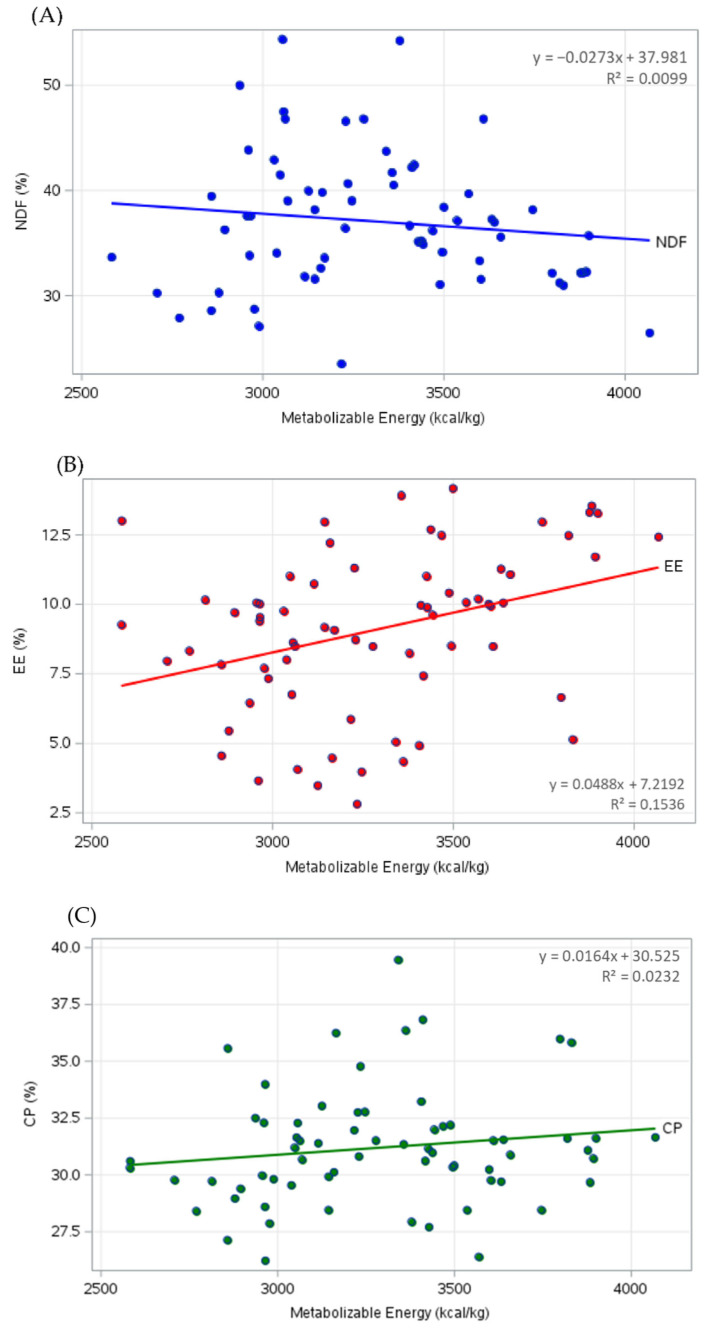
Relationship between metabolizable energy (ME) and nutritional components of corn distillers’ dried grains with solubles (DDGS) from 2010–2025. (**A**) Neutral detergent fiber (NDF), (**B**) ether extract (EE), and (**C**) crude protein (CP). Each point represents an individual observation, and the solid line represents the linear regression trend. The mean crude protein (CP) content was 31.20, the ether extract (EE) content was 9.07%, the neutra l detergent fiber (NDF) content was 37.08%, and the coefficients of variation were as follows: CP content, 8.13%; EE content, 31.97%; and NDF content, 17.32%; Number of observations: ME: 78, CP: 67, EE: 69, and NDF: 65.

The relationships between metabolizable energy and the nutritional constituents of corn DDGS are illustrated in Figure 2. Correlation analyses between metabolizable energy (ME) and the nutritional components of DDGS revealed low to moderate associations. As shown in Figure 2A, increasing ME levels were associated with a tendency toward lower neutral detergent fiber (NDF) concentrations (R^2^ = 0.0099). In contrast, the ether extract (EE) was positively related to the metabolizable energy (R^2^ = 0.1536) (Figure 2B). With respect to crude protein (CP), a slight increase in metabolizable energy was observed as CP levels increased (R^2^ = 0.0232) (Figure 2C).

These findings indicate that individual dietary components do not strongly influence the energy density of corn DDGS. In addition, the wide variability observed in the CP, EE, and NDF values suggests substantial differences in production and processing conditions.

**Figure 3:**
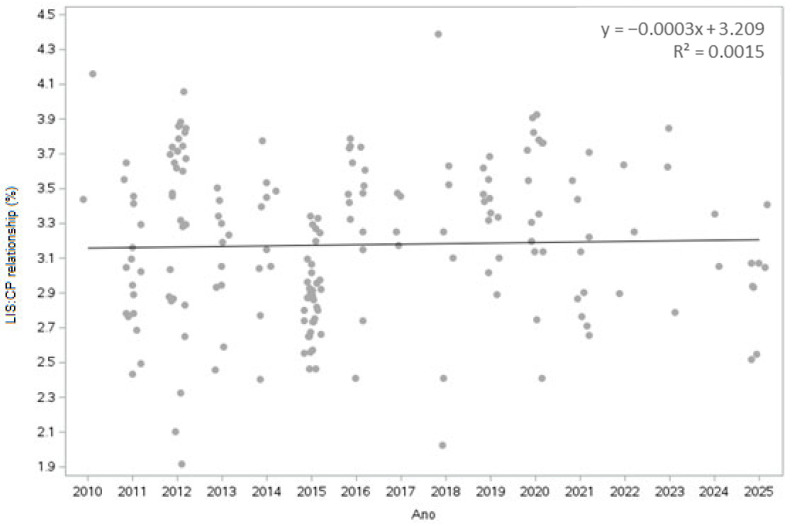
Lysine/protein ratio for 2010 to 2025 in corn DDGS. Mean: 3.21%; coefficient of variation: 20.88%; standard deviation: 0.67%; minimum: 1.10%; maximum: 7.47%. Model Adjustment: Nine outliers were removed to allow for better observation and visualization of the data, resulting in a total of 187 observations.

The lysine/protein ratio (Lys/CP) greatly varied over time. The trend line indicates that the Lys/CP ratio has a slight positive linear trend, indicating an increase in the Lys/CP ratio (Figure 3). Most of the values are between 2.5% and 4.0%, with some outliers.

To facilitate visualization and maintain the integrity of the data distribution, outliers were identified and removed only for the lysine/protein ratio data on the basis of the interquartile range (IQR) via the Statistical Analysis System in Demand for Academics software (Release 3.1.0, SAS Institute Inc., Cary, NC, USA).

The first quartile (Q1) and the third quartile (Q3) of the data distribution were calculated. The IQR was determined by the difference between Q3 and Q1. On the basis of this interval, the lower and upper limits were defined for the exclusion of outliers via Formulas (1)–(3):

(1) IQR=Question 3− Question 1

(2) Lower Limit=Q1−1.5×IQR

(3) Upper Limit=Q3+1.5×IQR

Observations with Lys/protein values outside this range were considered outliers and were excluded from the subsequent analysis.

## 4. Discussion

As expected, there is great variability in the composition and digestibility of corn ethanol products. Similar results were reported by Curry et al., Espinosa et al., and Paula et al. [6,7,10], demonstrating the variability of coproducts. Each plant, in order to improve or add value to the coproduct, uses different processes of milling, fermentation, drying, and drying temperature, in addition to opting for or not for the removal of oils, separation of fibers, and inclusion of solubles and/or additives. Another factor that can also cause this variability is the composition and type of grain used in its manufacture, which varies according to the region, fertilization, and genetics of the grain [9].

Even though the average chemical composition of wheat grains is characterized by relatively high crude fiber (CF) and neutral detergent fiber (NDF) contents [12], the levels of these components in wheat DDGS are lower than those observed in corn and sorghum DDGS. This comparison highlights that ethanol production and coproduct processing can markedly influence the final chemical composition of DDGS.

The average composition of the DDGS of corn observed in this study, in general, was greater than that reported in the main Brazilian reference food composition Rostagno et al. [13], differing only in the NDF contents, where the mean NDF content was lower in the present study. Comparing the composition of the corn DDGS of the present study to the values observed in the NRC, the means are similar to the levels of the medium-oil DDGS, differing only in the Ca and P contents, where the values identified in the present study are higher. The same behavior can be observed in the amino acid composition of maize DDGS, which is greater than that determined by Rostagno et al. and similar to the data observed in the NRC [12,13].

When the nutritional composition of corn DDGS reported in this study was compared with the values presented by Rostagno et al. [13], similar crude protein (CP) contents were observed, but higher digestible lysine and metabolizable energy (ME) values were found. These differences may be associated with the higher ether extract (EE) and lower neutral detergent fiber (NDF) contents identified in this systematic literature review.

Compared with those in the NRC [12], higher CP and digestible lysine contents were observed in the present study, whereas the ME values were slightly lower, possibly due to the higher average NDF content. Chemical components such as EE and NDF are indicators of increasingly digestible fractions in swine diets and are therefore commonly used as predictors of energy and amino acid digestibility.

The higher percentage of NDF found in sorghum DDGS than in the other sources reported in this study corroborates the results estimated by Corassa et al. [184]. The energy values of sorghum DDGS observed in the present study were higher than those reported by Corassa et al. [184], where the value determined was 4345 kcal/kg. The concentration of the chemical components of DDGS may be influenced by the variability in the composition of the grains used in the ethanol production process [12].

The inclusion of solubles in the coproduct may explain the higher protein content observed in high-protein DDGS (HPDDGS) than in high-protein DDG (HPDDG), representing an additional source of variation in production processes that influences the chemical composition of these ingredients.

In turn, the values found in the present study for the HPDDG are higher than those observed in the NRC [12], differing only in the CP content, where the means observed in the present study are lower. Compared with those of Rostagno et al. [13], the chemical composition of the present study is similar, with higher values of CF, NDF, and ADF and lower mean EE.

In general, compared with the other sources, corn DDGS had better digestibility coefficients. Similar results were reported by other authors, where corn DDGS showed better digestibility than sorghum DDGS did [184]. Higher levels of fibrous fractions, such as neutral detergent fiber (NDF) and acid detergent fiber (ADF), as well as antinutritional factors, such as tannins derived from sorghum grains, may limit amino acid digestibility in pigs, which is reflected in the lower digestibility coefficients observed for sorghum DDGS than for corn DDGS.

The apparent digestibility coefficients of amino acids for the HPDDG observed in this study ranged from approximately 50–75%; these data were similar to those determined by Paula et al. [10]. In contrast, the values were lower than those estimated by Motta et al. [11] and the NRC [12].

According to Rostagno et al. [13], the values reported for the ME and DE for the DDGS and HPDDG of corn are 2930 and 3123 kcal/kg and 3620 and 4060 kcal/kg, respectively, whereas in the present study, the ME and DE values ranged from 3307 and 3566 kcal/kg and 3375 and 3653 kcal/kg, respectively. Compared with those of the NRC [12], the ME and DE values observed for DDGS in this study are similar to those established by the NRC [12] for DDGS with medium oil content: 3582 kcal/kg for ME and 3396 kcal/kg for DE. For the HPDDG, the NRC [12] estimates of DE and ME are 4040 and 3732 kcal/kg, respectively, which are higher than the values reported in the present study.

Differences in the ethanol production processes used by industries may lead to variations in the levels of ether extract, crude protein, and fiber in DDGS. Consequently, they can promote variations in the DE and ME of the coproducts and, as a result, affect these levels in the formulated diet [187].

The ability of pigs to derive energy from feed, expressed as digestible or metabolizable energy, is closely related to the structural characteristics of organic molecules, with starch being more readily utilized than fibrous fractions. Consequently, the higher digestible energy (DE) and metabolizable energy (ME) values observed in corn DDGS may be attributed to greater starch concentrations and lower neutral detergent fiber (NDF) and acid detergent fiber (ADF) contents than those in sorghum DDGS (Table 1). Therefore, the use of coproducts with higher energy density may be advantageous in diets for lactating sows or post-weaning piglets.

The reduction in NDF with increasing ME levels (Figure 2A) suggests that fiber contributes significantly to the digestibility of energy in corn ethanol byproducts, where a lower proportion of fiber in the ingredient favors the efficiency of energy digestibility in pigs. These data are consistent with those reported in the literature, showing that dietary fiber negatively affects the digestibility of energy and nutrients in pigs, increasing fecal production and nutrient excretion [188].

The increase in ME content is directly linked to the increase in EE, since lipids play a significant role in the energy metabolism of animals (Figure 2B). The EE content of DDGS is an important variable because it is related to differences in pig growth performance [185]. Similar data were reported by Corassa et al. [186], with lower EE and higher NDF contents affecting the energy value of corn ethanol coproducts.

Protein levels were stable as the ME increased, demonstrating that, compared with lipids, protein does not contribute significantly to increased energy in pigs because of low energy density [12].

As a practical implication, ingredients with low energy values may limit their inclusion in diets for animal categories with high energy requirements, such as lactating sows, or alternatively require combination with higher-energy ingredients to achieve an appropriate dietary balance.

Correlation analyses between metabolizable energy (ME) and the nutritional components of DDGS revealed low to moderate relationships (Figure 2). Although a negative relationship between ME and NDF content was identified, it explained only a small proportion of the observed variability (R^2^ = 0.0099), indicating that NDF alone accounts for a limited share of the variation in metabolizable energy. In contrast, ether extract (EE) had a stronger positive association with ME, with an R^2^ of 0.1536, indicating that approximately 15% of the observed variation could be attributed to lipid content, making it the most influential nutritional component among those evaluated. Crude protein (CP) also exhibited a positive relationship with ME; however, the low coefficient of determination suggests that its isolated impact on metabolizable energy is limited. Therefore, these results indicate that the ME of DDGS is multidetermined and that a univariate assessment of individual nutrients provides limited explanatory power.

Finally, the predictive capacity of the models presented herein highlights the need for more accurate estimation of the ME of corn ethanol coproducts via multivariate approaches based on large datasets.

The lysine-protein ratio varied substantially over the analyzed period (Figure 3), with a coefficient of variation of 20.88%, which may directly affect pig performance when these coproducts are included in swine diets. However, despite the high variability, no consistent temporal trend was observed, as regression analysis across years yielded an extremely low coefficient of determination (R^2^ = 0.0015). This indicates that the year of publication explains less than 1% of the observed variation. These findings suggest that variations in the lysine-protein ratio are independent of time and are more closely associated with intrinsic factors related to ethanol processing conditions.

The industrial process of ethanol production can affect the content of amino acids present in DDGS, and processes such as the inclusion of soluble compounds, the addition of urea, cooking, yeast protein, and grain milling influence nitrogen compounds [189].

The mean lysine/protein ratio of the DDGS observed in the present study was 3.21%. Compared with data for soybean meals, an ingredient commonly used in the diets of production animals, this value is considered low. With respect to 45% soybean meals, the lysine/protein ratio is approximately 6.31% [13]. However, for solvent-extracted soybean meals, which contains 43.90% CP, the mean lysine/protein ratio is 6.29% [12].

Lysine is an essential amino acid and plays an important role in the synthesis of muscle tissue in pigs. Low lysine/protein values indicate a lower amount of lysine in relation to the total protein content in the diet, necessitating greater supplementation with synthetic amino acids to meet the nutritional requirements of the animals.

A lower amino acid supply or reduced amino acid digestibility in ethanol coproducts may limit their use in swine diets, particularly for more sensitive or nutritionally demanding categories, such as post-weaning piglets and lactating sows, respectively. In such cases, dietary formulation may require the inclusion of complementary ingredients, such as soybean meals or industrial amino acids, to correct these nutritional limitations.

The variability observed in this study across several nutritional composition parameters of grain ethanol coproducts raises concern throughout the entire production and utilization chain. This variability hampers precise diet formulation and may negatively affect productive and economic performance. Therefore, ethanol industries should seek to monitor and mitigate sources of variation to further standardize their coproducts, thereby increasing value and improving the overall viability of the production chain.

Despite the relevant findings of the present study, a limited number of data points were available for certain variables, such as the amino acid content of sorghum DDGS and the mineral content and metabolizable energy of wheat DDGS. Additionally, other potential sources of variation, including animal genetics, experimental protocols, and the use of feed additives, were not addressed in this systematic review. Consequently, the results and conclusions presented herein should be interpreted in light of these limitations.

Therefore, further analyses using this database are warranted to better elucidate the sources of variation in the nutritional composition of ethanol coproducts and to potentially expand the dataset to include additional types of information.

## 5. Conclusions

The composition of the corn ethanol coproducts varied across the years analyzed. The data provide a broad analysis of the variability in the composition and energy values of the coproducts, as well as their digestibility.

The collected data reinforce the importance of considering the great variability of corn ethanol coproducts for pig diet formulations.

## Figures and Tables

**Table 1:** DDGS compositions of different grains between 2010 and 2025.

Composition (%)	Corn (359)					Sorghum (15)					Wheat (32)				
	Mean	Min	Max	SD	N	Mean	Min	Max	SD	N	Mean	Min	Max	SD	N
DM ^1^	89.05	72.00	94.59	2.29	301	90.17	87.50	92.97	1.69	13	91.29	88.6	93.72	1.38	28
CP ^2^	30.56	14.51	50.84	3.29	325	31.49	25.77	39.13	4.24	14	36.28	26.94	46.3	4.69	31
GE (kcal/kg) ^3^	5015	3295	6261	448	205	4812	4345	5295	364	9	4953	4514	5479	234	14
EE ^4^	8.97	1.24	17.96	3.07	256	9.26	5.80	11.73	1.44	14	6.51	3.9	13.64	2.39	29
Starch	7.45	0.19	25.62	4.86	142	4.37	2.50	7.75	1.86	6	3.43	0.51	12.9	2.72	19
CF ^5^	9.95	2.65	31.17	4.78	97	8.75	5.89	12.35	2.21	8	7.79	5.2	10.41	1.37	21
NDF ^6^	35.51	13.96	54.99	6.34	273	40.84	28.24	66.10	13.70	12	31.53	22.74	42.7	5.02	30
ADF ^7^	12.53	3.55	25.6	3.11	258	24.31	14.69	40.94	7.51	10	14.03	6.86	27.37	5.38	26
MM ^8^	5.19	1.25	9.07	1.05	232	5.06	3.32	9.98	0.07	8	5.30	3.75	9.3	0.93	28
Minerals (%)															
Ca	0.10	0.01	2	0.19	127	0.18	0.06	0.57	0.22	5	0.11	0.01	0.18	0.05	7
P	0.81	0.25	1.19	0.18	160	0.68	0.45	0.87	0.17	5	0.82	0.11	1.05	0.33	7
Cu	0.003	0.0005	0.016	0.004	16	0.01	0.01	0.01	0.001	3	-	-	-	-	-
Fe	0.02	0.006	0.074	0.02	16	0.12	0.12	0.13	0.01	3	-	-	-	-	-
Mg	0.29	0.045	0.36	0.07	15	0.35	0.23	0.42	0.10	3	-	-	-	-	-
Mn	0.004	0.0001	0.05	0.01	16	0.04	0.03	0.04	0.004	3	-	-	-	-	-
K	1.19	1.01	1.47	0.11	32	0.95	0.54	1.17	0.35	3	1.34	-	-	-	1
I	0.0002	-	-	-	1	-	-	-	-	-	-	-	-	-	-
Na	0.23	0.04	0.51	0.09	32	-	-	-	-	-	0.25	-	-	-	1
S	0.76	0.01	1.39	0.35	28	0.58	0.42	0.77	0.18	3	-	-	-	-	-
Zn	0.016	0.0005	0.05	0.02	16	0.04	0.04	0.04	0.004	3	-	-	-	-	-
Essential amino acids (%)															
Arg	1.26	0.12	2.61	0.27	196	1.20	1.15	1.30	0.07	4	1.97	1.06	4.67	1.12	14
Phe	1.47	0.43	2.69	0.24	181	1.30	-	-	-	1	2.31	1.03	5.01	1.34	13
His	0.82	0.28	1.48	0.14	195	0.68	0.62	0.73	0.02	4	1.04	0.62	2.44	0.61	13
Ile	1.11	0.00	2.14	0.24	203	1.29	1.16	1.52	0.11	7	1.81	1.13	4.23	1.03	14
Leu	3.42	0.43	5.88	0.71	202	3.71	3.27	4.60	0.47	7	3.42	2.14	7.46	1.82	14
Lys	0.96	0.23	1.99	0.20	213	0.91	0.81	1.12	0.10	7	0.99	0.52	2.33	0.50	18
Met	0.57	0.01	1.15	0.12	205	0.51	0.43	0.62	0.06	7	0.75	0.44	1.67	0.39	15
The	1.11	0.35	1.99	0.17	209	1.04	0.94	1.14	0.07	7	1.49	0.94	3.45	0.80	16
Trp	0.22	0.12	0.89	0.08	191	0.22	0.16	0.28	0.04	6	0.48	0.26	1.11	0.28	10
Val	1.51	0.54	3.00	0.25	203	1.65	1.49	1.83	0.11	7	2.26	1.40	5.23	1.26	14
Nonessential amino acids (%)															
Ala	2.13	0.51	3.60	0.33	180	2.25	-	-	-	1	1.95	1.12	3.9	1.02	13
Asp	1.90	0.60	3.57	0.32	180	1.86	-	-	-	1	2.54	1.58	5.35	1.38	13
Cys	0.56	0.27	1.05	0.09	180	0.51	0.45	0.58	0.05	4	0.93	0.36	2.34	0.55	15
Glu	4.05	0.49	7.95	1.02	179	3.75	-	-	-	1	13.40	5.93	31.4	8.35	13
Gly	1.16	0.47	2.17	0.19	178	1.12	-	-	-	1	2.05	1.24	4.23	1.13	13
Pro	2.35	0.34	3.72	0.44	169	1.93	-	-	-	1	4.63	2.68	11.02	2.75	13
Ser	1.81	0.01	4.97	1.13	180	1.16	-	-	-	1	2.28	1.32	5.57	1.42	13
Tyr	1.07	0.24	2.01	0.26	173	0.84	-	-	-	1	1.90	1.02	3.56	1.08	8

^1^ Dry matter; ^2^ Crude protein; ^3^ Gross energy; ^4^ Ether extract; ^5^ Crude fiber; ^6^ Neutral detergent fiber; ^7^ Acid detergent fiber; ^8^ Mineral matter. The values are expressed as the means, minimums (Mins), and maximums (Máx), followed by standard deviations (SDs) and the number of observations (N). Hyphen (-) indicates data not reported in the original studies.

**Table 2:** Composition of the HPDDG and HPDDGS between 2010 and 2025.

	HPDDGS Corn (14)					HPDDG Corn (31)				
Composition (%)	Mean	Min	Max	SD	N	Mean	Min	Max	SD	N
DM ^1^	90.34	86.50	93.90	1.97	12	92.51	87.45	98.59	3.23	29
CP ^2^	42.50	36.25	54.78	5.06	13	42.23	36.55	60.21	6.89	31
GE (kcal/kg) ^3^	5040	4142	5578	417	8	5446	5011	5840	227	12
EE ^4^	8.81	3.07	16.71	4.09	11	8.20	1.97	12.50	2.86	29
Starch	3.72	1.09	8.73	4.34	3	3.06	2.51	4.10	0.89	3
CF ^5^	8.07	4.10	12.30	4.34	3	10.99	4.88	28.10	6.44	10
NDF ^6^	33.19	27.10	48.34	5.75	13	39.90	30.00	52.97	6.85	26
ADF ^7^	16.29	12.57	22.90	2.97	9	20.69	10.30	30.00	6.07	16
MM ^8^	2.43	1.84	2.85	0.36	6	2.90	1.38	8.60	1.65	16
Minerals (%)										
Ca	0.09	0.01	0.50	0.15	9	0.06	0.01	0.20	0.06	19
P	0.49	0.04	0.91	0.29	9	0.52	0.04	0.79	0.23	19
Essential amino acids (%)										
Arg	1.75	1.37	2.02	0.21	11	1.82	1.45	2.51	0.24	23
Phe	2.29	2.01	2.81	0.22	11	2.28	1.89	3.59	0.48	23
His	1.18	1.00	1.56	0.17	11	1.20	0.99	1.60	0.19	23
Ile	1.74	1.48	2.19	0.21	11	1.76	1.43	2.49	0.32	22
Leu	5.63	4.78	7.69	0.90	11	5.42	3.95	9.31	1.42	23
Lys	1.33	1.05	1.65	0.21	11	1.29	0.98	1.87	0.24	23
Met	0.88	0.79	1.12	0.10	11	0.97	0.60	1.44	0.22	23
Thr	1.59	1.34	1.91	0.16	11	1.55	0.14	2.13	0.40	23
Trp	0.26	0.11	0.35	0.07	9	0.78	0.14	4.47	1.27	23
Val	2.24	1.92	2.85	0.27	11	2.33	1.94	2.99	0.32	23
Nonessential amino acids (%)										
Ala	3.29	2.78	4.45	0.56	10	3.48	2.94	5.08	0.69	15
Asp	2.79	2.35	3.15	0.27	10	3.06	2.42	3.81	0.45	15
Cys	0.83	0.73	0.92	0.07	10	0.94	0.77	1.18	0.14	15
Glu	7.09	6.10	8.16	0.63	10	7.75	6.25	10.92	1.54	15
Gly	1.94	1.26	3.60	0.93	10	1.70	1.31	2.19	0.25	15
Pro	3.14	1.65	4.21	0.92	9	4.00	3.24	5.44	0.72	13
Ser	1.99	1.60	2.36	0.29	10	2.06	1.65	2.57	0.33	15
Tyr	1.81	1.51	2.20	0.22	8	1.89	1.40	2.87	0.43	15

^1^ Dry matter; ^2^ Crude protein; ^3^ Gross energy; ^4^ Ether extract; ^5^ Crude fiber; ^6^ Neutral detergent fiber; ^7^ Acid detergent fiber; ^8^ Mineral matter. The values are expressed as the means, minimums (Mins), and maximums (Máx), followed by standard deviations (SDs) and the number of observations (N).

**Table 3:** Digestibility coefficients and energy content of DDGS from different grains between 2010 and 2025.

	Corn (359)					Sorghum (15)					Wheat (32)				
Composition (%)	Mean	Min	Max	SD	N	Mean	Min	Max	SD	N	Mean	Min	Max	SD	N
DM ^1^	64.79	15.70	83.49	14.90	34	69.25	66.60	71.90	3.75	2	68.20	66.00	70.40	3.11	2
CP ^2^	71.44	44.00	92.20	10.08	89	63.43	51.30	72.20	10.85	3	67.78	51.87	90.00	18.41	4
EE ^3^	66.45	21.80	97.90	14.41	27	59.10	57.90	60.30	1.69	2	97.00	-	-	-	1
Starch	99.60	99.50	99.70	0.14	2	-	-	-	-	-	-	-	-	-	-
CF ^4^	12.59	9.90	16.33	2.02	8	13.60	-	-	-	1	-	-	-	-	-
NDF ^5^	55.59	21.10	73.80	12.93	51	65.17	60.70	70.40	4.90	3	-	-	-	-	-
ADF ^6^	46.60	5.47	84.80	27.87	35	13.83	-	13.83	-	1	-	-	-	-	-
MM ^7^	43.75	16.80	60.00	20.85	6	54.30	53.30	55.30	1.41	2	79.00	-	-	-	1
GE ^8^	67.09	28.00	78.60	13.46	27	66.55	63.20	69.90	4.73	2	75.00	-	-	-	1
Energy content (kcal/kg)															
GE ^8^	4946	2371	6261	601	211	4812	4345	5295	364	9	4953	4514	5479	232	14
DE ^9^	3566	2850	5086	348	86	3349	3030	3520	195	5	-	-	-	-	-
ME ^10^	3307	2581	4087	350	81	3156	2863	3300	194	5	-	-	-	-	-

^1^ Dry matter; ^2^ Crude protein; ^3^ Ether extract; ^4^ Crude fiber; ^5^ Neutral detergent fiber; ^6^ Acid detergent fiber; ^7^ Mineral matter; ^8^ Gross energy; ^9^ Digestible energy; ^10^ Metabolizable energy. The values are expressed as the means, minimums (Mins), and maximums (Máx), followed by standard deviations (SDs) and the number of observations (N). Hyphen (-) indicates data not reported in the original studies.

**Table 4:** Digestibility coefficient and digestible amino acid content of DDGS from different grains between 2010 and 2025.

	Corn (331)					Sorghum (15)					Wheat (32)				
Digestible Content	Mean	Min	Max	SD	N	Mean	Min	Max	SD	N	Mean	Min	Max	SD	N
Essential amino acids (%)															
Arg	0.96	0.64	2.14	0.25	43	0.75	-	-	-	1	0.89	0.81	1.03	0.12	3
Phe	1.11	0.77	2.22	0.24	42	0.72	-	-	-	1	1.03	1.00	1.06	0.04	2
His	0.63	0.37	1.20	0.17	43	0.38	-	-	-	1	0.68	0.60	0.73	0.07	3
Ile	0.80	0.12	1.71	0.24	43	0.71	-	-	-	1	0.80	0.70	0.96	0.14	3
Leu	2.76	1.98	4.90	0.52	43	1.93	-	-	-	1	1.94	1.48	2.81	0.75	3
Lys	0.60	0.28	1.62	0.31	43	0.68	-	-	-	1	0.24	0.08	0.54	0.26	3
Met	0.46	0.29	0.95	0.11	41	0.29	-	-	-	1	0.37	0.30	0.49	0.11	3
Thr	0.74	0.43	1.55	0.25	43	0.50	-	-	-	1	0.59	0.49	0.79	0.17	3
Trp	0.17	0.05	1.11	0.19	30	-	-	-	-	0	1.57	1.56	1.58	0.01	2
Val	1.03	0.12	2.32	0.31	43	0.92	-	-	-	1	0.97	0.85	1.20	0.20	3
Nonessential amino acids (%)															
Ala	1.57	1.04	2.79	0.37	39	1.25	-	-	-	1	0.87	0.53	1.55	0.59	3
Asp	1.19	0.008	2.62	0.47	39	0.83	-	-	-	1	0.85	0.62	1.30	0.39	3
Cys	0.39	0.25	0.84	0.13	37	0.18	-	-	-	1	0.41	0.37	0.49	0.07	3
Glu	3.21	1.59	6.55	1.00	39	2.01	-	-	-	1	6.83	5.57	8.36	1.55	3
Gly	0.65	0.01	2.83	0.40	39	0.40	-	-	-	1	0.50	0.37	0.75	0.21	3
Pro	1.43	0.12	2.41	0.49	24	0.77	-	-	-	1	1.92	-	-	-	1
Ser	1.26	0.61	3.58	0.88	39	0.59	-	-	-	1	1.03	0.93	1.18	0.13	3
Tyr	0.82	0.59	1.66	0.18	37	0.61	-	-	-	1	0.85	-	-	-	1
Digestibility coefficient of essential amino acids (%)															
Arg	76.38	59.20	87.13	7.26	50	62.70	-	-	-	1	72.06	66.08	82.30	9.74	3
Phe	78.42	63.86	87.52	5.31	50	60.50	-	-	-	1	74.51	68.20	86.40	10.30	3
His	73.29	62.18	85.80	5.87	50	57.90	-	-	-	1	69.55	62.86	82.60	11.30	3
Ile	73.11	57.41	97.50	7.36	50	61.00	-	-	-	1	69.32	62.70	82.00	10.98	3
Leu	82.22	68.25	90.31	4.57	50	63.10	-	-	-	1	75.03	68.09	88.30	11.50	3
Lys	55.76	27.60	83.10	11.80	52	66.20	-	-	-	1	33.83	15.91	64.30	26.53	3
Met	81.04	67.31	90.60	4.79	48	68.80	-	-	-	1	72.51	64.64	86.50	12.14	3
Thr	63.81	41.00	81.30	8.17	50	48.60	-	-	-	1	58.70	49.83	75.50	14.56	3
Trp	64.19	44.00	86.40	11.43	43	-	-	-	-	0	53.09	51.54	54.64	2.19	2
Val	70.84	56.00	83.70	6.43	50	56.70	-	-	-	1	66.60	59.24	80.10	11.71	3
Digestibility coefficient of nonessential amino acids (%)															
Ala	75.07	63.81	87.70	6.04	47	60.80	-	-	-	1	56.28	42.55	82.80	22.97	3
Asp	63.61	46.00	79.80	7.91	47	48.70	-	-	-	1	50.34	38.32	72.90	19.55	3
Cys	66.97	46.40	83.00	8.16	45	44.50	-	-	-	1	67.26	61.53	78.30	9.57	3
Glu	77.50	61.87	88.80	5.53	47	58.80	-	-	-	1	87.23	80.91	91.99	5.70	3
Gly	47.13	1.00	79.69	17.38	47	39.50	-	-	-	1	39.32	28.25	60.60	18.43	3
Pro	59.04	5.60	96.67	22.28	33	43.60	-	-	-	1	71.80	-	-	-	1
Ser	72.84	50.76	94.03	9.69	47	55.70	-	-	-	1	72.01	64.79	85.00	11.27	3
Tyr	80.02	61.38	92.60	5.68	45	79.50	-	-	-	1	82.90	-	-	-	1

The values are expressed as the means, minimums (Mins), and maximums (Máx), followed by standard deviations (SDs) and the number of observations (N). Hyphen (-) indicates data not reported in the original studies.

**Table 5:** Digestibility coefficients and energy content of DDGS of the HPDDG and HPDDGS between 2010 and 2025.

	HPDDGS Corn (14)					HPDDG Corn (31)				
Composition (%)	Mean	Min	Max	SD	N	Mean	Min	Max	SD	N
DM ^1^	60.03	48.40	72.53	9.91	4	-	-	-	-	-
CP ^2^	64.93	52.30	77.00	12.35	3	59.39	34.00	74.80	13.11	8
EE ^3^	-	-	-	-	-	-	-	-	-	-
Starch	-	-	-	-	-	-	-	-	-	-
CF ^4^	-	-	-	-	-	-	-	-	-	-
NDF ^5^	58.85	38.70	75.00	25.66	2	-	-	-	-	-
ADF ^6^	-	-	-	-	-	-	-	-	-	-
MM ^7^	52.00	-	-	-	1	-	-	-	-	-
Energy content (kcal/kg)										
GE ^8^	5045	4142	5578	450	7	5446	5011	5840	227	12
DE ^9^	3628	3590	3667	54.44	2	3653	2267	4405	879	5
ME ^10^	3545	3330	3698	192	3	3375	2166	4070	743	5

^1^ Dry matter; ^2^ Crude protein; ^3^ Ether extract; ^4^ Crude fiber; ^5^ Neutral detergent fiber; ^6^ Acid detergent fiber; ^7^ Mineral matter; ^8^ Gross energy; ^9^ Digestible energy; ^10^ Metabolizable energy. The values are expressed as the means, minimums (Mins), and maximums (Máx), followed by standard deviations (SDs) and the number of observations (N). Hyphen (-) indicates data not reported in the original studies.

**Table 6:** Digestibility coefficient and digestible amino acid content of the HPDDG and HPDDGS between 2010 and 2025.

	HPDDGS Corn (14)					HPDDG Corn (31)				
Digestible Content	Mean	Min	Max	SD	N	Mean	Min	Max	SD	N
Essential amino acids (%)										
Arg	1.18	0.89	1.57	0.28	4	0.88	0.58	1.50	0.27	12
Phe	1.49	1.40	1.55	0.07	3	0.97	0.66	1.58	0.30	12
His	0.80	0.62	1.19	0.26	4	0.48	0.33	0.89	0.17	12
Ile	1.07	0.94	1.19	0.10	4	0.71	0.44	1.24	0.25	12
Leu	4.12	3.74	4.55	0.33	4	2.14	0.17	4.13	1.09	12
Lys	0.66	0.47	1.04	0.25	4	0.68	0.19	1.03	0.23	12
Met	0.65	0.60	0.69	0.03	4	0.45	0.36	0.74	0.11	12
Thr	0.87	0.73	1.11	0.16	4	0.58	0.38	1.11	0.21	12
Trp	0.60	0.04	1.17	0.79	2	0.11	0.04	0.20	0.06	4
Val	1.36	1.21	1.56	0.14	4	0.89	0.61	1.67	0.33	12
Nonessential amino acids (%)										
Ala	2.19	1.92	2.52	0.30	3	1.85	1.48	2.38	0.40	4
Asp	1.68	1.41	2.05	0.33	3	0.94	0.01	2.23	1.11	4
Cys	0.53	0.45	0.61	0.08	3	0.51	0.23	0.84	0.24	4
Glu	5.48	4.92	6.16	0.62	3	4.56	3.49	6.21	1.17	4
Gly	1.05	0.33	2.29	1.07	3	0.35	0.07	0.93	0.39	4
Pro	1.13	0.69	1.57	0.62	2	-	-	-	-	0
Ser	1.38	1.16	1.70	0.28	3	1.10	0.83	1.53	0.31	4
Tyr	1.42	-	-	-	1	1.06	0.87	1.41	0.24	4
Digestibility coefficient of essential amino acids (%)										
Arg	74.40	59.60	87.40	12.63	5	71.92	42.00	92.60	15.08	8
Phe	75.95	69.50	90.40	9.80	4	72.79	50.00	93.10	12.19	8
His	72.44	60.90	87.50	11.73	5	69.26	42.00	92.00	14.75	8
Ile	73.28	62.90	88.30	11.38	5	66.62	40.00	92.10	14.49	8
Leu	83.42	75.90	92.00	6.95	5	76.81	57.00	94.80	11.23	8
Lys	56.08	39.20	76.20	16.84	5	51.64	17.00	87.10	19.77	8
Met	81.68	73.30	92.40	8.01	5	77.10	55.00	94.90	11.57	8
Thr	64.50	50.50	84.50	14.56	5	60.81	29.00	88.40	16.43	8
Trp	69.30	37.90	90.50	27.74	3	58.52	33.00	90.00	17.97	8
Val	72.22	60.40	87.20	11.60	5	65.55	36.00	91.10	15.24	8
Digestibility coefficient of nonessential amino acids (%)										
Ala	79.53	69.10	90.30	9.62	4	70.16	48.00	93.40	13.07	8
Asp	67.90	53.70	84.30	13.48	4	62.16	31.00	88.50	15.99	8
Cys	72.60	61.70	86.00	10.44	4	64.81	29.00	90.30	17.44	8
Glu	82.38	73.60	90.90	8.16	4	74.26	54.00	94.00	11.61	8
Gly	52.58	22.70	78.80	26.78	4	38.41	5.00	86.20	26.86	8
Pro	49.47	21.10	81.00	30.08	3	58.99	33.72	88.90	18.59	6
Ser	74.40	61.40	88.50	12.45	4	69.41	46.00	90.60	14.13	8
Tyr	88.50	86.50	90.50	2.83	2	73.96	58.00	93.40	10.46	8

The values are expressed as the means, minimums (Mins), and maximums (Máx), followed by standard deviations (SDs) and the number of observations (N). Hyphen (-) indicates data not reported in the original studies.

## Data Availability

The raw data supporting the conclusions of this article will be made available by the authors without undue reservation.
